# Clinical use of Dieletrophoresis separation for live Adipose derived stem cells

**DOI:** 10.1186/1479-5876-10-99

**Published:** 2012-05-17

**Authors:** Allan Y Wu, David M Morrow

**Affiliations:** 1 The Morrow Institute, 69-780 Stellar Drive, Rancho Mirage, CA, 92270, USA

## Abstract

**Background:**

Microelectrode dieletrophoresis capture of live cells has been explored in animal and cellular models *ex-vivo*. Currently, there is no clinical data available regarding the safety and efficacy of dielectrophoresis (DEP) buffers and microcurrent manipulation in humans, despite copious pre-clinical studies suggesting its safety. The purpose of this study was to determine if DEP isolation of SVF using minimal manipulation methods is safe and efficacious for use in humans using the hand lipotransfer model.

**Methods:**

Autologous stromal vascular fraction cells (SVF) were obtained from lipoaspirate by collagenase digestion and centrifugation. The final mixture of live and dead cells was further processed using a custom DEP microelectrode array and microcurrent generator to isolate only live nucleated cells. Lipotransfer was completed using fat graft enhanced with either standard processed SVF (control) versus DEP filtered SVF (experimental). Spectral photography, ultrasound and biometric measurements were obtained at post operatively days 1, 4, 7, 14, 30, 60 and 90.

**Results:**

The DEP filter was capable of increasing SVF viability counts from 74.3 ± 2.0% to 94.7 ± 2.1%. Surrogate markers of inflammation (temperature, soft tissue swelling, pain and diminished range of motion) were more profound on the control hand. Clinical improvement in hand appearance was appreciated in both hands, though the control hand exclusively sustained late phase erosive skin breaks on post operative day 7. No skin breaks were appreciated on the DEP-SVF treated hand. Early fat engraftment failure was noted on the control hand thenar web space at 3 months post surgery.

**Discussion:**

No immediate hypersensitivity or adverse reaction was appreciated with the DEP-SVF treated hand. In fact, the control hand experienced skin disruption and mild superficial cellulitis, whereas the experimental hand did not experience this complication, suggesting a possible “protective” effect with DEP filtered SVF. Late ultrasound survey revealed larger and more frequent formation of oil cysts in the control hand, also suggesting greater risk of engraftment failure with standard lipotransfer.

**Conclusion:**

Clinical DEP appears safe and efficacious for human use. The DEP microelectrode array was found to be versatile and robust in efficiently isolating live SVF cells from dead cells and cellular debris in a time sensitive clinical setting.

## Background

Automated devices for stem cell processing and isolation are of great clinical utility to the growing field of bioengineering and regenerative cellular therapy. Adipose derived stem cells (ADSC) in particular have come under greater use and scrutiny in regenerative medicine due to: relative ease of access from liposuction [1], abundance compared to other tissues [2] and lack of controversy [3]. Heterogeneous populations of ADSCs are commonly obtained from the stromal vascular fraction (SVF) of processed adipose tissue. Currently there are commercial devices capable of isolating the SVF [4], however no clinical machines to date are capable of separating live versus dead cells. This becomes a particular problem for immediate clinical cellular therapy in that concomitant injection of dead cells contributes to inflammation thereby reducing odds of cell engraftment and transplantation [5]. Moreover, cellular debris induced inflammation could pose the further threat of inhibiting normal differentiation of ADSC [6]. Removal of dead cells could be easily achieved with tissue culture incubation and further purification steps, however, some regulatory agencies, such as the United States Food and Drug Administration, forbid the use of concomitant cell culture for cellular therapy to stay within the required limits of “minimal manipulation” for immediate clinical use [7].

Flow activated cytometry cell separation (FACS) or magnetic antibody cell separation (MACS) have also been proposed as a solution, yet these too are fraught with difficulties of finding FDA approved good manufacturing process (GMP) antibodies [8]. Other technical concerns are antibody mediated activation of markers, which in some cases are actually receptors or mediators of transduction pathways affecting cell behavior [9]. Additionally, the process further requires “washing and removing” antibodies before human use. For all the above reasons, FACS and MACS in sum are expensive, prohibitively time consuming and technically challenging beyond the scope of routine clinical practitioners. A minimally manipulated, facile, non-labeled and cost efficient method of harvesting live cells would be of tremendous value to the growing regenerative medicine field.

**Figure 1:**
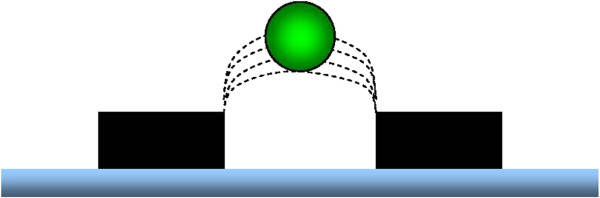
Schematic drawing of microelectrodes (black rectangles) trapping a stem cell (green sphere) within a DEP field (dotted lines) in the absence of any laminar flow across the array.

**Figure 2:**
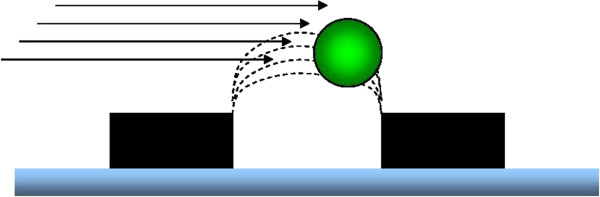
**Schematic drawing of `microelectrodes (black rectangles) trapping a stem cell (green sphere) within a DEP field (dotted lines) in the presence of laminar flow (black arrows) across the array.** The cell is displaced laterally to the trailing edge of the DEP field.

Cell separation by dielectrophoresis (DEP) could provide a method of fulfilling the economic, logistical and regulatory criterion laid out above. In simple terms, DEP cell isolation is a method of trapping charged spheroid particles such as free floating live human cells in a low conducting medium over a micro-electrode. The micro-electrode in turn is connected to an oscillating current generator at specific functional frequencies. Interestingly, dead cells do not express or exhibit a surface charge, as cellular ion channels are no longer active. Dead cells can therefore essentially be washed through a DEP device, while live cells may be captured by a field of the dielectric Force (F_DEP_) created by an array of micro-electrodes (Figures 1 and 2) [10]. 

The “capture” force (F_DEP_) exerted on a charged cell in this clinical context may be defined mathematically as:

(1) $${\text{F}}_{\text{DEP}}=2\pi {\text{R}}^{3}\varepsilon_{1}\left\{\text{Re}\left[\text{K}\left(w\right)\right]\right\}\nabla {\text{E}}^{2}$$

In simple terms this means that F_DEP_ is related to size (πR^3^) and polarizability (2Re[K(*w*)]) of the cell, permittivity (ε_1_) of the suspension medium and gradient of the electric field (∇E^2^) applied to the device. By manipulating these parameters it is possible to either trap or repulse cells at will [11].

Historically DEP found initial use in the biotech industry for massive-scale tissue culture processing systems [12]. Only recently has DEP been utilized to experimentally isolate discrete cell types such as: blood [13], marrow [14], spermatozoa [15], oocytes [16], ADSC and neuronal progenitor cells [17]. While DEP has not been shown to detrimentally affect viability of cells in culture [18], DEP isolated cells have never been utilized for direct human use in any clinical application at the time of this publication. To simply assume *in-vitro* data can be extrapolated to human use may not be correct, since intermittent exposure to high residual electro-oxidized carbohydrates may ultimately diminish tissue engraftment and SVF function *in-vivo*[19,20]. Therefore, the purpose of this study is to determine if DEP isolated SVF is comparable in safety and efficacy to conventional SVF obtained by simple centrifugation in the hand atrophy model.

## Methods

### Consents

Written consent was obtained for the procedure in addition to consent for enrollment into a clinical trial. Consent forms and experimental protocol were reviewed and unanimously approved by an independent IRB (FWA #A00013119). Consent and procedure was performed at a State sanctioned hospital in Mexico cleared for therapeutic autologous stem cell therapy by the Subsecretaría de Regulación y Fomento Sanitario Secretaría de Salud of the United Mexican States.

### Construction of device

**Figure 3:**
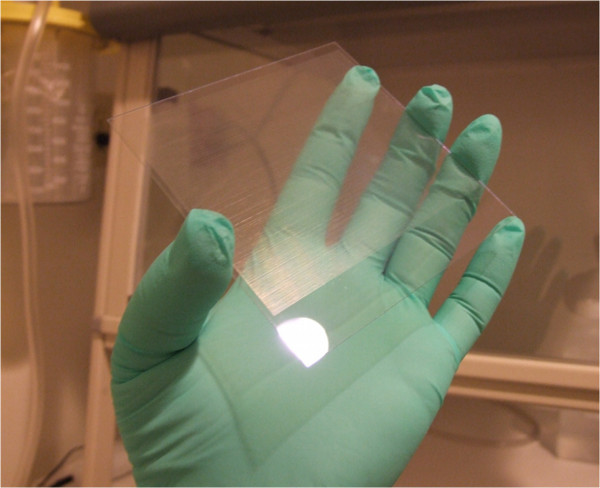
Photograph of DEP array prior to sterile packaging into clinical cartridge.

The dielectrophoresis filter was constructed from ITO conductive glass and etched by a high precision laser to produce an inter-digitating microelectrode pattern in a planar array (Figure 3), and further modified to tolerate high concentration processing without cell clumping or parasitic F_DEP_ “traps”. The microelectrode width was 50 microns, whereas the gap space between electrodes was 100 microns. The microelectrode was then sandwiched between custom ¼ inch Plexiglas plates with a 0.2 mm PDMS gasket to form a sealed space above the planar array. An alternating signal generator to allow a dynamic range of 2 kHz to 1 MHz and a V_p-p_ maximum of 15 volts was custom manufactured specifically for this clinical application. Waveforms were confirmed real time using a Tektronix® TDA640 oscilloscope throughout cell isolation steps. The entire filter was mounted on an inverted microscope stage to observe capture real time.

### DEP isolation parameters

A low ionic, high carbohydrate media supplemented with antioxidants was used as manipulation buffer for the device. Conductivity was adjusted to 30 micro Sieverts and the buffer was sterile filtered (0.45 micron 43052 Corning, Corning NY). The generator was set within a frequency range from 100 to 1,000 kHz and the chamber flow rate through the filter regulated at 2 cc/min. A soak cycle of 10 minutes was allowed prior to final specimen collection. Cells were washed and centrifuged twice in sterile normal saline. Final pellet was resuspended in 2.5 cc of sterile normal saline prior to being admixed with purified fat.

### Tissue collection and processing

To minimize risk of “wet mess” contamination, routine ice bagging for preliminary regional anesthesia was avoided in favor of skin cooling with dry ice blocks wrapped in a custom sterile thermal restrictive material, which limited cooling to 4^o^ Celsius. All procedures, tissue and cell handling were carried out at room temperature. Infiltration and liposuction was performed using standard Klein tumescent solution with a three-hole 4 mm bullet tip cannula. Vacuum extraction was regulated at a negative pressure maximum of 350 mmHg throughout the procedure. A total of 400 cc of fat and 600 cc infranatant was obtained. Only washed fat was used for the study (infranatant was discarded). A total of 6 × 10^8^ viable SVF cells were isolated from a 200 cc aliquot of fat, which were pelleted and resuspended in a volume of 5 cc of DEP buffer. Following DEP processing, 7 × 10^6^ viable nucleated cells were obtained and admixed into washed, but not collagenase treated, fat for cell assisted lipotransfer.

Fat was washed in a closed sterile system using sterile normal saline serially x3. Half of the washed fat was reserved for later processing. The other half was treated with collagenase enzyme (Custom Compounding, Los Alamitos CA) to dissociate the SVF from the adipose tissue [21]. Pelleted SVF cells were then suspended in 25 cc of normal saline, of which half was used for standard therapy and the remaining further processed using the experimental device under isolation parameters stated above. Control SVF was incubated for the same amount of time with the same amount of DEP buffer as the experimental protocol. Both control and experimental SVF were washed, centrifuged, repelleted and reconcentrated to 1 × 10^6^ cells/mL in endotoxin free plastic ware (BT 1024, 1210; BP 1003-B Biomed Resource, Riverside CA, http://www.bmres.com). A small 100 uL aliquot sample was obtained from both SVF suspensions for cellular assays prior to being mixed back with purified non-digested fat. Fat and SVF was mixed by gentle nutation for 20 minutes prior to injection.

### Viability

In a sub-experiment we evaluated the effects of saline, DEP buffer and presence of an applied electric field on SVF viability using matching time intervals and frequencies to that of the clinical protocol. The test sample aliquot was divided evenly into three sets: 1. saline control 2. DEP buffer 3. DEP buffer with applied current and 4. DEP buffer with applied current in “live capture” mode with a dead cell washout. Samples were perfused into separate but equivalent microelectrode array cartridges and eluted from the chamber using matched suspension fluids. All cells were collected and processed for Trypan blue dye exclusion. The experiment was replicated times three the same day and counted by the same observer.

### Flow cytometry

Aliquots of pre- and post-DEP treated cells were analyzed by Guava-PCA flow cytometry using ViaCount(R) (Millipore 4000–0040 LOT 11–0115) reagent or monoclonal anti-human CD34 directly conjugated to phycoerythrin (R&D Systems FAB7227P LOT: ACOG01) according to manufacturer protocol. A minimum of 10,000 events were counted for each sample. Flow data was analyzed by Guava ExpressPlus software (Millipore). For CD34 expression, total counts were corrected by subtracting cells reactive to isotype control. CD34+ cells were then enumerated as a percentage of corrected total counts.

### Lipotransfer

Hand atrophy correction is an experimental model well suited for this study for the following reasons: 1. two sites (left or right hand) may be used for either control or experimental treatment whereby 2. the patient functions as their own internal control and 3. minimal fat is required for harvest and processing.

One month prior to surgery patient was tested for hypersensitivity to experimental buffer with a 1.0 cc wheal beneath the skin on the antecubitum. No reaction was appreciated and anergy panel testing was negative. Enhanced fat grafts using either control SVF or DEP treated (experimental) SVF were transferred using a 2 mm blunt tip cannula with a single side port. Entry into the subdermal space was made by a single 16’ needle puncture at the wrist to allow passage of the transfer cannula. The patient and surgeon were blinded to the status (experimental vs. control) of the graft. Enhanced fat graft was distributed by serial fanning method using minimal pressure on withdrawal to deploy a thin ribbon of graft while avoiding vessels and tendinous sheaths of the hand. Over correction by 20% was performed bilaterally as this is customary standard of care. Immediate post operative photo documentation was obtained. Steri-strips® were used to close puncture sites and hands were wrapped in loose Kerlex gauze.

### Standardization of photography

Of great concern in photographing patients being treated for hand atrophy is visual biasing secondary hand and digit position. For example: fully extending the digits on a preoperative photograph will accentuate a skeletonized appearance, whereas non-extension in the post-operative photograph will appear near normal. In some patients this biased positioning is enough to create the false appearance of atrophy correction even without surgical intervention. For this reason we developed a standardized method of photographing the hands as follows:

1. the patient is placed in the sitting position facing a blue/green photography wall, such that the hips are flexed at 90 degrees and knees touching the wall,

2. arms are fully extended directly forward to the patient

3. palms are placed on wall creating 45 degree angle between plane of ground and arms

4. inter-hand distance is to be no wider than the width between shoulders

5. digits are then photographed in the following positions:

a. fully extended,

b. closed with thenar at 90 degrees relative to other digits

6. multispectral images for detailed skin evaluation are obtained by Visia® (Canfield, Fairfield NJ), an automated photodocumentation system, with the hand in a closed fist position and touching the top reference bar.

### Biometric measurements

Patient was measured for extension range of motion in her wrist and digits using specialty goniometers pre- and post-operatively, with the entire antecubetum held flush with the table. Circumference was taken at the base of each digit with a finger circumference gauge. Average circumference was calculated by taking the sum of each digit for each hand and dividing by 5. Thermocouple readings were performed on the center dorsum of the had at room temperature (Digisense® Thermocouple, Cole-Palmer, Bunker CT). Readings were obtained after 3 minutes of equilibration with the metal lead.

## Results

### Case description

Case #72-001: Patient was a 69-year-old Caucasian female (BMI 29) and reformed smoker experiencing repetitive skin breaks echymosses and trauma to dorsum of hands secondary to thinning of the skin. She was also unsatisfied with the skeletonized appearance on the dorsum of her hands. Old scars (hypopigmented hyperkeratotic lesions) from inadvertent abrasions and ruptured skin from traumatic echymosses were appreciated on pre-operative evaluation. One year of routine high emollient topical skin therapy was unsuccessful. Recurrent ease of trauma began to affect her ability to carry out normal hand function and activities of daily living. Conventional dermal fillers were also offered as temporizing therapy, however, the patient reported a history (three years previous) of an adverse reaction to a dermal filler, thus a preference for autologous therapy. For this reason she requested definitive therapy with autologous fat transfer. After unblinding at the conclusion of the study (3 months following the procedure) a CA-125 (5 U/mL) and AFP (2 ng/dL) were drawn from the experimental hand and found to be negative. (Negative range for our reference laboratory is 35 U/mL for CA-125 and 10 ng/dL for AFP.)

### DEP effects on viability

**Figure 4:**
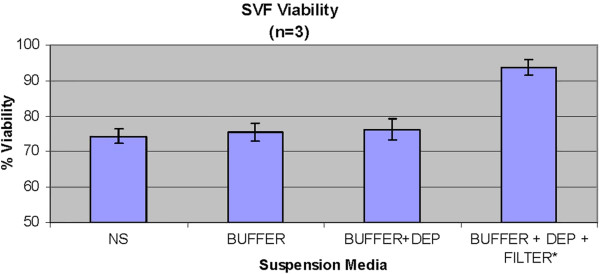
Percent viability measured by Trypan dye exclusion versus type of buffer and/or presence of DEP field.

Simple perfusion of SVF suspended in normal saline through the device cartridge produces a baseline viability of 74.3 ± 2.0% (Figure 4). Buffer without an electric field yielded 75.4% viability, whereas microelectric current exposure with buffer in whole cell collection mode (i.e. no washout of dead cells) slightly increased the viability to 76.2 ± 3.1%. Buffer with device in live capture mode dramatically increased viability to 94.7 ± 2.1%.

### DEP effects on biometry readings

**Figure 5:**
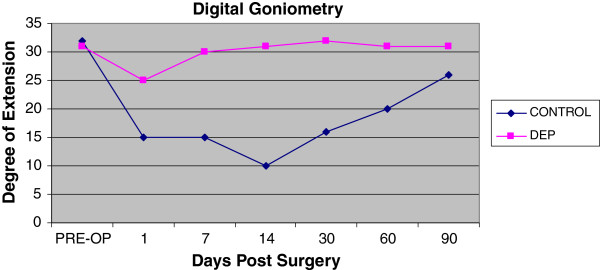
Goniometry measurements with fingers in the maximally extended position.

**Figure 6:**
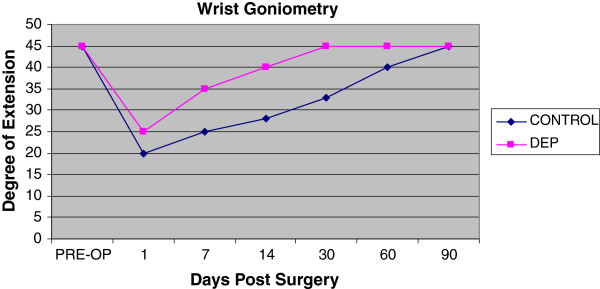
Goniometry measurements of wrist in maximal extension position post surgery.

**Figure 7:**
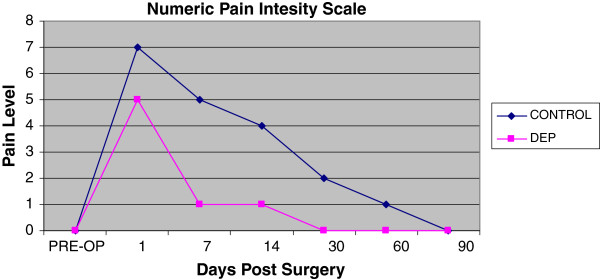
Numeric self reported pain scale levels post surgery.

**Figure 8:**
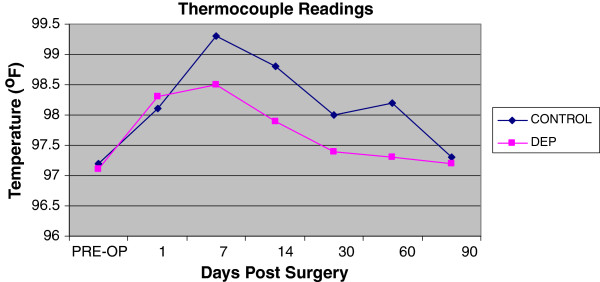
Thermocouple surface readings at center of hand on the dorsum surface.

**Figure 9:**
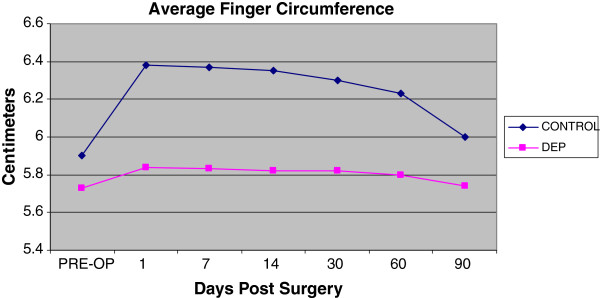
Average finger circumference measured as a function of time post surgery.

Both treatment and control hand experienced a temporary decrease in extension range of motion of the digits and wrist. Of interesting note, the digits on the control hand did not have full return to baseline at the end of 3 months (Figure 5). The control hand also had a slower rate of return to baseline in goniometry compared to the DEP treated hand (Figures 5 and 6). Pain also contributed to the limitation of extension motion and was worse in the control hand even at rest with NSAID pain medication (Figure 7). Thermocouple readings from the center of the hand also revealed localized temperature elevations in both hands, but a higher Tmax (99.3°F) for the control hand and a slower return to baseline compared to DEP treated (Figure 8). Thermal elevation appeared 7 days post surgery whereas a goniometry, pain and finger circumference appeared earlier at 1 day post surgery (Figures 5, 6, 7, 8 and 9). 

### Flow cytometry data

**Figure 10:**
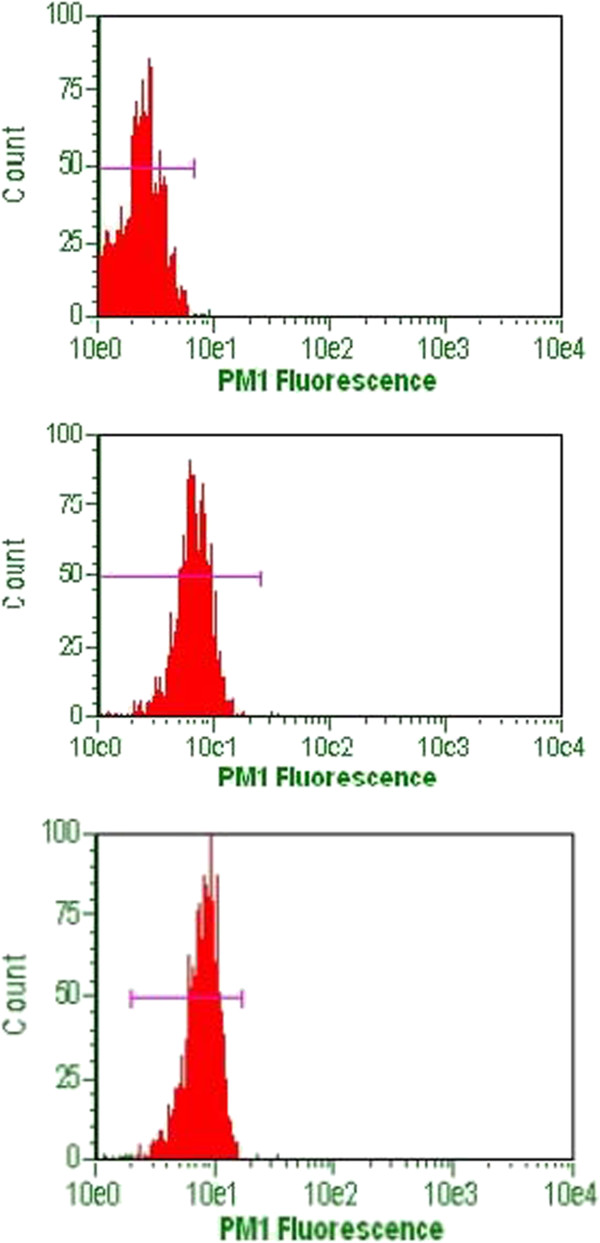
Flow cytometry for detection of CD34 cells in DEP isolated SVF (lower panel) versus non-treated SVF (middle panel) and background control (upper panel).

**Figure 11:**
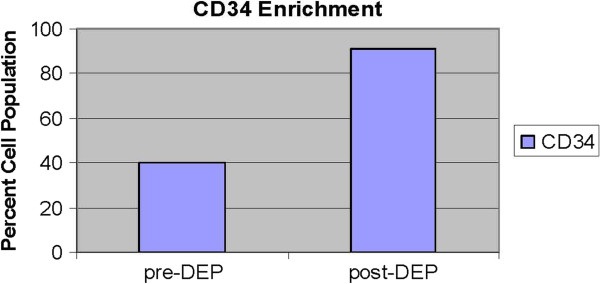
CD34 cell counts expressed as a percentage cell population determined by flow cytometry, before and after DEP isolation.

**Figure 12:**
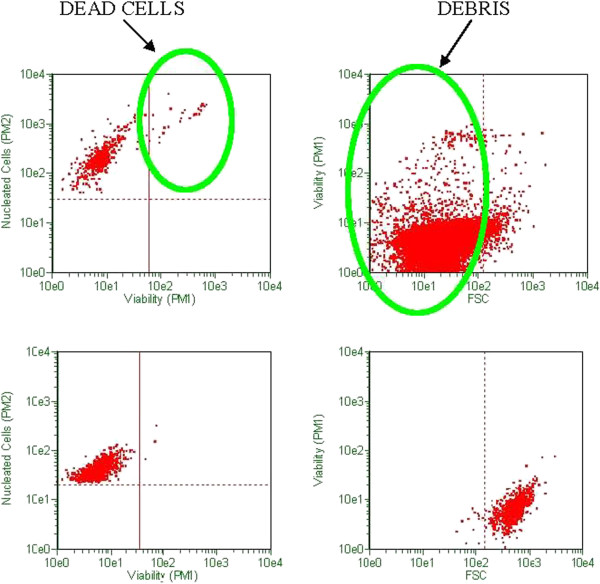
**Viability and debris analysis by flow cytometry using ViaCount stain.** High dead cell and debris (green circles) appreciated in un-treated SVF (upper panels). DEP isolated and “cleaned” SVF (lower panels).

**Figure 13:**
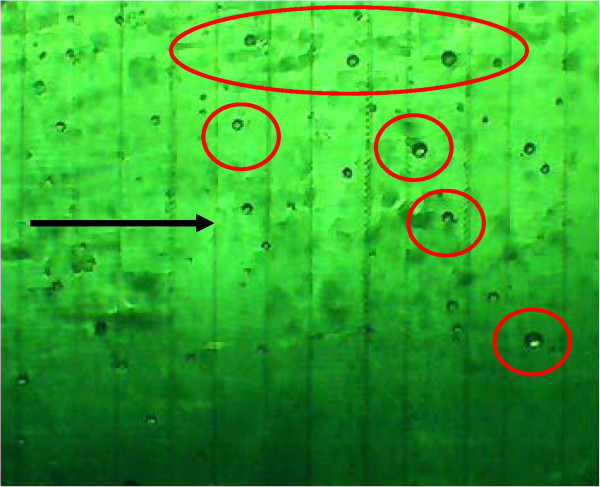
**Microscopic view (4x inverted microscope phase and green filter) of DEP microelectrode array using higher frequencies to capture smaller nucleated cells (red circles) 25–40 microns in size within a high debris clinical sample.** Darker striped regions are the electrode domains (approximately 50 microns) whereas the lighter striped regions are the gap space (approximately 100 microns). The direction of flow (black arrow) causes some captured cells to be displaced to the trailing edge of the DEP field.

**Figure 14:**
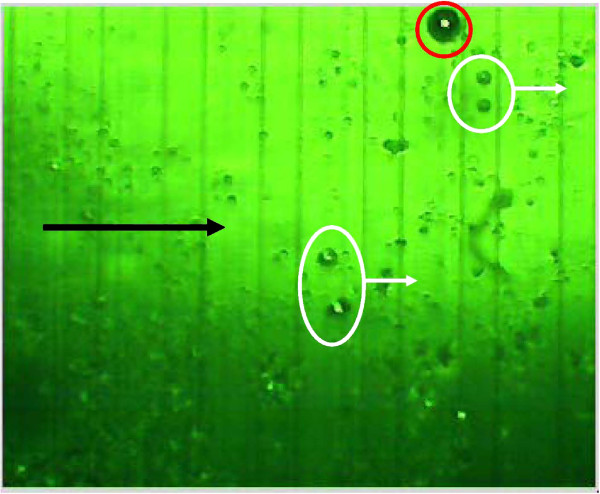
**Microscopic view (4x inverted microscope phase and green filter) of DEP microelectrode array using a different modulating capture frequency to collect 50 micron cells (red circle) trapped lateral to the microelectrode (dark stripe), despite heavy debris field.** Smaller debris and cells (white circles) flow through the chamber and are washed free from the captured cells.

Viability and CD34 counts are improved with DEP isolation of SVF (Figures 10 and 11). A “cleaning” effect to the cell population is appreciated after DEP isolation, in that dead cells and cellular debris are cleared from the sample (Figures 12, 13 and 14). A more narrow homogeneous profile and distribution of fluorescence is also appreciated on forward scatter for CD34 staining in DEP isolated cell populations (Figure 10). Untreated cells exhibit a bimodal distribution, whereas DEP isolated cells have a single peak and narrow bell shaped distribution with considerably higher CD34 counts. An increase in CD34 levels improved from 41% to 90%, which is a greater than two-fold increase following DEP isolation (Figure 11). 

## Discussion

**Figure 15:**
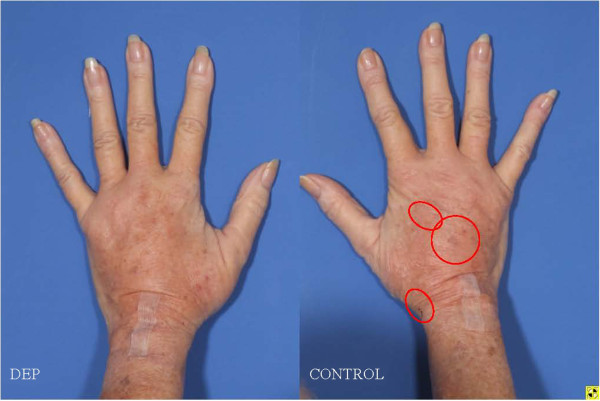
**69-year-old female status post hand lipotransfer post operative day 7 with DEP treated (left pane) versus control (right pane).** Note eruptions, ulcers and skin breaks (red circles) which occurred exclusively on the control hand. Swelling is more prominent in the control (right) hand, but erythema is noted in both.

**Figure 16:**
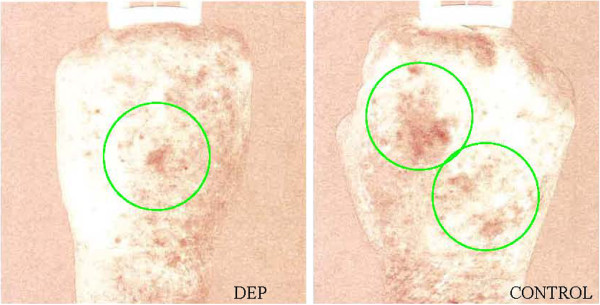
**69-year-old female status post hand lipotransfer procedure post operative day 4.** Spectral imaging of hands with RBX mode showing regions of inflamed skin (green circles) with DEP (left panel) versus control (right panel). Regions of ulceration and skin breaks occurred in the control hand within the inflamed regions, yet no skin breaks or ulcers occurred at any point of the study in the DEP treated hand.

**Figure 17:**
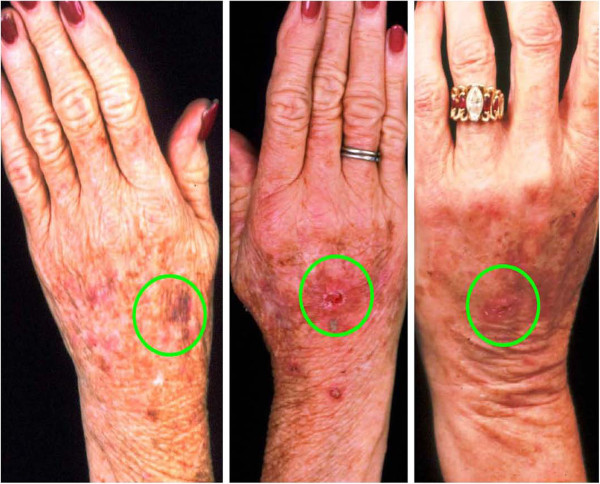
**A cohort of hands in women aged 60-73-years-old within 1 week post standard lipotransfer.** The typical complication revealing ulceration and eruptions (green circle) within the center of the hand.

**Figure 18:**
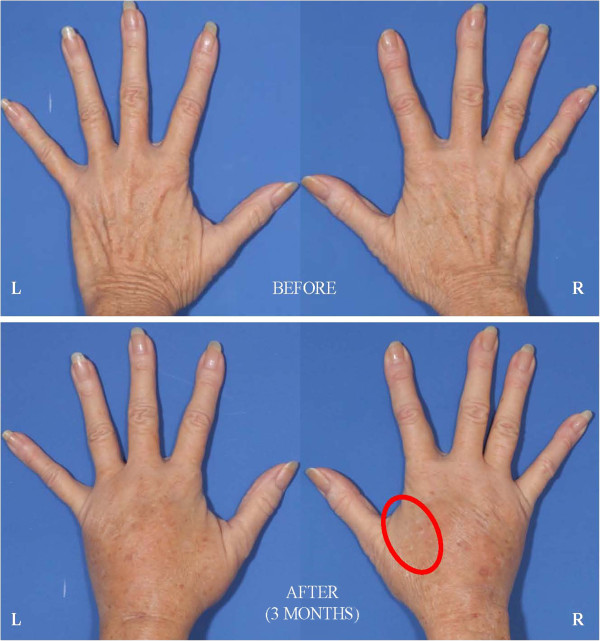
**69-year-old female status post hand lipotransfer 3 months post surgery.** Before and after images of hands treated with standard fat transfer (right hand) versus DEP purified SVF (left side). Note early loss of engraftment noted in control hand thenar web space (red circle).

To our knowledge, this is the first reported clinical use of DEP processing of SVF cell therapy in a human. Contrary to our original hypothesis, a significant improvement in clinical outcomes was appreciated with the experimental procedure of the study. Positive benefit was noted in immediate post-surgical healing parameters (Figures 5, 6, 7, 8 and 9). Of particular note: post-surgical skin eruptions and ulcerations occurred only on the control hand (Figure 15). Location of skin breaks were highly correlated to RBX filter images showing vascular injection and dilation occurring towards the center of the hand (Figure 16). This is consistent with other fat transfers we have performed with and without SVF supplementation in the past (Figure 17). Interestingly, the experimentally treated hand sustained vascular changes as well, but to a smaller surface area, yet did not have any skin breaks or ulcerations despite receiving equal volumes of enriched fat graft. Moreover, the side with DEP purified SVF exhibited superior engraftment especially in the largest volume treatment region, the thenar web space, which is a common site of failure (Figure 18 lower panels). 

**Figure 19:**
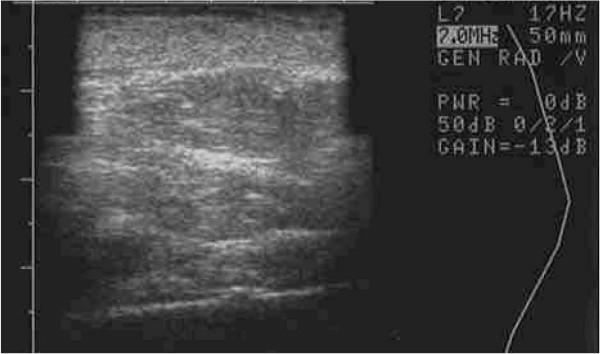
Ultrasound image of left hand (DEP-SVF treated) saggital view of thenar web space of a 69-year-old female 3 months post lipotransfer.

**Figure 20:**
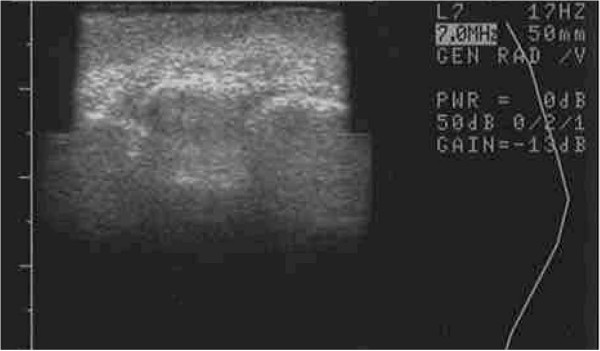
Ultrasound image of left hand (DEP-SVF treated) coronal view of thenar web space of a 69-year-old female 3 months post lipotransfer.

**Figure 21:**
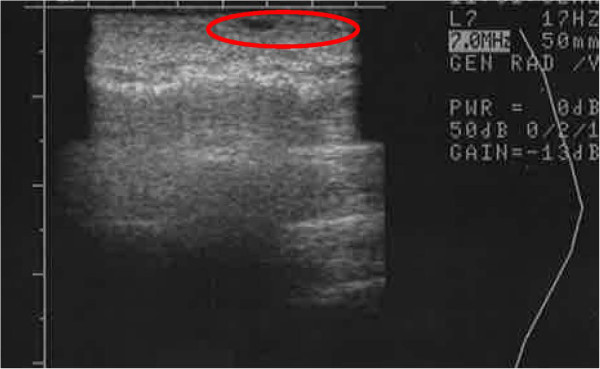
**Ultrasound image of right hand (control) saggital view of thenar web space of a 69-year-old female 3 months post lipotransfer.** Oil cysts seen in superficial dermis (red circle).

**Figure 22:**
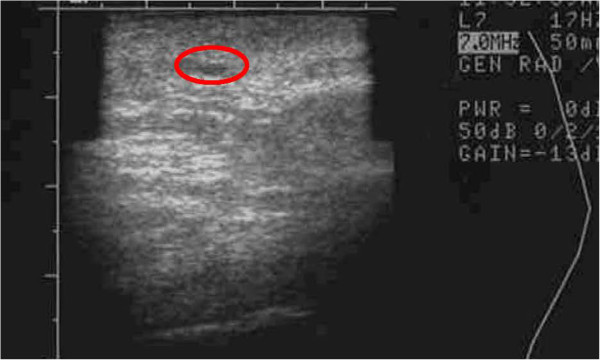
**Ultrasound image of right hand (DEP-SVF treated) coronal view of thenar web space in a 69-year-old female 3 months post lipotransfer.** Oil cyst seen in deeper dermis (red circle).

It is not entirely clear why a clinical difference occurred, though great efforts were taken to control for similar conditions (i.e. equivalent cell handling and DEP media exposure for both interventions). Measures were also taken to select a patient with similar appearing hands and endogenous vascularization confirmed by arterial Doppler flow studies prior to therapy. Both interventions from a clinical perspective, however, did yield comparable appearing aesthetic results, though the skin consistency of the DEP-SVF treated hand was palpably more dense with normal turgor, whereas the control hand retained a parchment like quality to the skin (Figure 18). Further differences in deeper subdermal consistency were also appreciated on ultrasound in the control hand, such that oil cysts (an early sign of graft failure) were appreciated in greater number than the DEP-SVF treated hand (Figures 19, 20, 21 and 22). 

**Figure 23:**
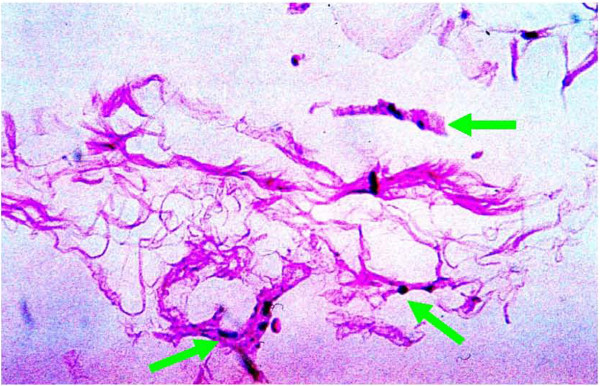
**Microscopic image (40X, H and E staining) of fibrillary tangles of cellular debris and adipocyte matrix biopsied from a hand 3 weeks post standard fat transfer.** (Previous patient not part of this study.) Note mast cells (green arrows) surrounding cellular debris.

**Figure 24:**
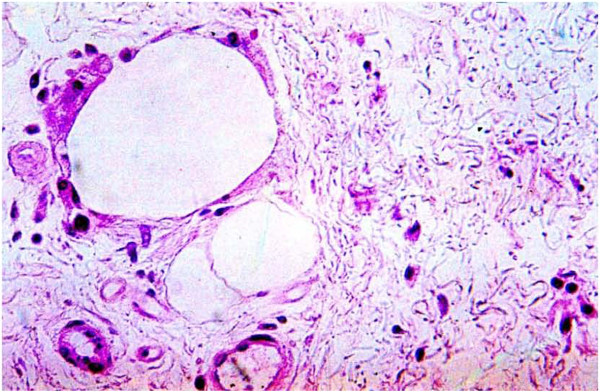
**Microscopic image (40X, H and E staining) of engrafted fat with dense fibrosis (white arrow) surrounding nascent fat globules (black arrow) at 6 months post standard lipotransfer.** (Previous patient not part of this study).

**Figure 25:**
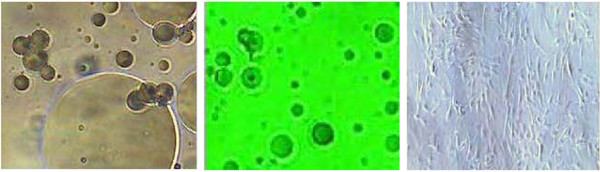
**40x microscopic image of SVF before (left pane) and after (middle pane) DEP isolation.** (Smaller dark particles in middle panel are unsettled defocused SVF cells.) DEP isolated cells directly plated to Matrigel coated culture dish (right panel) without mechanical filtration at 10x magnification (note absence of large adipocytes and cellular debris).

Unexpectedly the patient sustained a minor decrease in extension capability at the wrist and digits in the control hand. Though minor, any functional deficit can have an exponential effect on an already compromised dexterity, due to concomitant disease (i.e. arthritic joint disease and tendon contractures). We have speculated in 30 years of performing standard lipotransfer of the hand that, in select cases, cellular debris may contribute to surrounding fibrillary tangles (Figure 23) and eventual fibrosis (Figure 24) surrounding tendon sheaths, which impairs normal free movement of the hand. Perhaps this complication may be related to cellular focusing of SVF cells (Figure 25) and the elimination of cellular debris (Figures 12, 13 and 14) and inflammatory mediators, which recruit adipose lipophages and other inhibitory cells to exacerbate endogenous underlying disease (i.e. arthritis) [5]. This is suggested indirectly by the higher thermocouple readings (Figure 8) and other surrogate markers for inflammation (i.e. finger circumference, pain and decreased mobility) seen in the control hand. However, exposure to a DEP field in and of itself could also play a beneficial role by inducing cell cycle activation of “pre-viable” or quiescent (G_0_) cells, which in a typical transfer provide no immediate healing functions. Further studies to disentangle these issues in addition to randomized control trial studies with the DEP device are currently being conducted at our centers. 

Though current beneficial implications of accelerated healing and improved engraftment are promising, clinical DEP presents even greater future implications for the field of regenerative medicine and cell therapy. F_DEP_ can be modulated in direction (i.e. capture vs. repulsion) and magnitude. The methodology is also versatile enough to function at the nano- , micro- and macro- level. This affords the ability to “tune-in” or “tune-out” specific cell types from the heterogenous composition of SVF, which is unprecedented and represents the development of a second generation tool for regenerative medicine.

A device, such as the one used in this study, could be used to economically select or subtract cell types to more precisely define and refine cellular therapies. For example, breast augmentation using autologous SVF enriched adipose tissue has progressed with clinical results which continue to improve [22-26], but safety issues such as calcification artifacts interfering with mammogram screening and the long-term risks of tumorgenesis have been raised [27]. Additionally, recent epidemiologic data on prosthetic breast implants suggest foreign bodies within the breast space may be related to anaplastic large cell lymphoma (ALCL) as well [28]. Clinical DEP could provide a safer breast fat graft by removal of osteogenic (calcification forming) precursors [29] or CD-30 (ALCL associated) lymphocytes, which coincidentally can occur in high abundance as a contaminant in SVF (unpublished findings). Furthermore, DEP is also capable of detecting high nuclear to cytoplasmic ratio cells and extremely small charged particles, affording the possibility of “filtering” any graft free of cancer cells [30,31] or bacteria [32]. DEP can even select based on cell cycle status, opening the further possibility of improving cell therapy transplantation rates by transferring only actively dividing cells [33].

Adipose SVF and ADSC have also found use in sports medicine, orthopedics and rehabilitation therapy. One particular treatment showing strong demand is treatment of damaged or worn cartilage with SVF/ADSC. There are conflicting reports of efficacy for this indication [34]. Inadvertent simultaneous transplantation of pre-committed adipose precursor cells within the SVF into articular spaces could have poor long-term consequences. Recurrent damage of engrafted ectopic fat on articular surfaces release lipid and long chain fatty acids. This can be converted into prostaglandins and other mediators of inflammation (i.e. adipokines), thus accelerating native cartilage degeneration [35]. Joint space injections with purified chondrocyte destined cells, minus adipogenic precursors, would be preferable to a random heterogeneous approach currently in use and presents yet another example of how clinical DEP could be applied.

In more recent developments, popular media reports of regenerative medicine utilization by prominent athletes (i.e. Peyton Manning, Chad Ochocinco and Terrell Owens) have created a glamorizing effect to the field. While these famous cases of stem cell therapy are helpful in raising awareness, disproportionate attention to the promise, but not the potential consequences, leave serious concerns of the public being unfairly biased and indirectly counseled by dominating modern media and content [36]. Though it is not the intention of this paper to morally assail any practitioner or patient, we believe the growing demand for cellular therapies is reaching critical mass and signifies the necessity of a clinical paradigm shift from a focus of efficacy, to one of safety. To this end, all second generation separation technologies, especially DEP, should be investigated with a greater sense of urgency to address the growing immediate need for safety.

## Conclusions

Though only one human subject was used for this pilot study, five independent injections were performed for each hand web space and no long-term sonographic irregularities (hyperechoic or hypoechoic) were appreciated with DEP processing. Clinical DEP processing of SVF cells, therefore, appears to be feasible and safe for use in humans, and confers an improved healing and engraftment capability in comparison to standard SVF in the hand lipotransfer model. Use of DEP in the clinical setting offers the promise of a potentially more safe, rational and personalized approach to cellular therapeutics.

## Competing interests

The authors declare they have no competing interests.

## Authors’ contributions

AW carried out design and construction of apparatus, clinical studies and completed flow cytometric studies and analysis. DM participated in apparatus and clinical study design and provided original clinical background data. Both authors read and approved the final manuscript.
