# Expression of concern: Clinical use of dieletrophoresis separation for live adipose derived stem cells

**DOI:** 10.1186/s12967-014-0297-8

**Published:** 2014-11-04

**Authors:** Francesco M Marincola

**Affiliations:** Sidra Medical and Research Center, Doha, Quatar

After publication of this article [1], it was brought to the journal’s attention that there were potentially several serious concerns regarding the reliability of the data presented within it.

There is uncertainty regarding the timeline shown in figures 5 to 9 and the timing of the images shown in figures 15 and 16 and 18 to 22. Allegations have also been made that the experiments may not have taken place and that informed consent may not have been obtained.

Despite extensive efforts to obtain an independent investigation, we have been unable to verify these claims or come to a definitive conclusion regarding the reliability of the data. Readers are therefore urged to take caution when interpreting the content of this article.

Readers are alerted that an erratum has also been published regarding the authorship of this article [2].
